# Sound-Assisted Fluidization for Temperature Swing Adsorption and Calcium Looping: A Review

**DOI:** 10.3390/ma14030672

**Published:** 2021-02-01

**Authors:** Federica Raganati, Paola Ammendola

**Affiliations:** Istituto di Scienze e Tecnologie per l’Energia e la Mobilità Sostenibili (STEMS)—CNR, P.le V. Tecchio, 80125 Napoli, Italy; federica.raganati@stems.cnr.it

**Keywords:** sound-assisted fluidization, cohesive fine/ultra-fine particles, Carbon Capture and Storage (CCS), CO_2_ adsorption/desorption, temperature swing adsorption (TSA), thermochemical energy storage (TCES), calcium looping

## Abstract

Fine/ultra-fine cohesive powders find application in different industrial and chemical sectors. For example, they are considered in the framework of the Carbon Capture and Storage (CCS), for the reduction of the carbon dioxide emissions to the atmosphere, and in the framework of the thermochemical energy storage (TCES) in concentrated solar power (CSP) plants. Therefore, developing of technologies able to handle/process big amounts of these materials is of great importance. In this context, the sound-assisted fluidized bed reactor (SAFB) designed and set-up in Naples represents a useful device to study the behavior of cohesive powders also in the framework of low and high temperature chemical processes, such as CO_2_ adsorption and Ca-looping. The present manuscript reviews the main results obtained so far using the SAFB. More specifically, the role played by the acoustic perturbation and its effect on the fluid dynamics of the system and on the performances/outcomes of the specific chemical processes are pointed out.

## 1. Introduction

Fine and ultra-fine powders, i.e., characterized by particle size lower than 30 μm, have been characterized by increased research interest in recent years. In fact, due to their exceptional properties, mainly derived from their characteristic dimensions and large surface area per unit mass (which means that they are able to offer larger contact/reaction efficiency), they can be applied in a variety of industrial sectors, such as in the production of foods, plastics, cosmetics, catalysts, and metallurgic components [1].

For instance, nanostructured catalysts, characterized by a hierarchical porosity, have been synthesized in the framework of different chemical processes, such as combustion and gasification and fuel and polymer production [2]. Likewise, fine/ultrafine particles have also found application in the Carbon Capture and Storage (CCS) sector, for the reduction of the CO_2_ emissions to the atmosphere [3,4], and in the thermochemical energy storage (TCES) in concentrated solar power (CSP) plants [5,6,7,8].

In regards to the CCS, different separation techniques are available to separate the CO_2_ from the combustion flue gases: absorption, adsorption, cryogenics separation, membranes, etc. [9,10,11]. Among these, amine-based absorption, even though being the most mature technological option [12,13], is characterized by several shortcomings, when applied for post-combustion capture: huge energy demand for the regeneration of the sorbent, thermal/chemical degradation of the amines, amine losses due to evaporation and corrosion problems [12]. Therefore, adsorption has been proposed as one of the most attractive and promising alternatives, being characterized by relatively low energy consumption for the sorbent regeneration, high selectivity, no liquid waste streams, and quite a wide range of possible operating temperatures [14,15,16,17]. In particular, regarding the critical issue of the sorbent regeneration [18,19,20,21], different strategies can be adopted, acting either on the pressure (PSA—pressure swing adsorption) or on the temperature (TSA—temperature swing adsorption) of the system to induce the desorption of the CO_2_ molecules [22,23,24,25]. In this framework, TSA combined with an indirect heating process (for example by means of heat exchanger tubes), is considered to be very promising for post-combustion operations [21,24,26]. However, the selection of the sorbent is crucial for adsorption to become a competitive solution [27,28,29,30]. Indeed, a good sorbent should be relatively convenient from the economic point of view, but, at the same time, it should be able to provide good performances at low CO_2_ pressure (<0.2 atm [23]): high equilibrium adsorption capacity, fast kinetics of adsorption/desorption, stability to cyclic operations and tolerance to water vapor and other possible impurities in the exhaust stream [31]. In the framework of the need to produce specific adsorbent materials, fine/ultrafine particles have attracted growing interest in research [32]. Indeed, they can quite easily serve as the substrate for the production of ad-hoc synthesized sorbents, i.e., characterized by remarkable affinity towards the molecules of CO_2_ [27,28,29]. As a matter of fact, due to their distinctive properties, size, and nature their surface chemistry and porous structure can be easily adjusted/functionalized at the molecular level, i.e., by introducing chemical groups/ligands with basic functionalities (e.g., carbonates, amino groups, etc.) able to tailor their adsorption behavior towards the CO_2_ molecules [27,28,29,33,34,35,36]. Besides that, it must be considered that most of the sorbents commercially available are produced in a fine powdered form [27,28,29].

Likewise, fine/ultrafine powders have also been proposed as a possible solution to overcome the drawbacks of the calcium looping (CaL) process, which can be carried out for both CCS [37] and TCES-CSP applications [7,8], according to the following reaction scheme:

(1) $${\mathrm{CaCO}}_{3}\;(s)\;⇄\;\mathrm{CaO}\;(s)\;+\;{\mathrm{CO}}_{2}(g)\;\;\;\Delta H_{r}^{0}=178\;\mathrm{kJ}/\mathrm{mol}.$$

In the former case, CaO/CaCO_3_ is used as sorbent material to capture/release CO_2_ by means of carbonation/calcination cycles [37]. In the latter case, CaO is used as an energy intensive material for the storage of energy in a chemical form by means of the reversible carbonation/calcination reaction [7,8]. In regards to the carbonation reaction, it must be underlined that it takes place in two successive stages: (i) a fast initial stage controlled by the reaction kinetics and by the transport of the CO_2_ molecules from the bulk phase to the particle surface and (ii) a slow final stage controlled by the diffusion of the CO_2_ molecules through the carbonate layer, which covers the remaining free surface of the CaO particles [38]. In light of this, it is clear that a poor gas–solid contact efficiency can strongly hinder the carbonation reaction [37]. Moreover, regardless of the specific application, i.e., either CCS or TCES, the main shortcoming of CaL is represented by the loss of CaO activity, i.e., the progressive drop of the carbonation conversion over repeated cycles due to sintering and pore-plugging [39,40]. Therefore, thanks to the extensive research effort devoted to finding solutions to this issue in the last years, different options are available to limit the natural CaO deactivation, such as: thermal activation [41,42], hydration [43], mechanical crushing [44,45], and the synthesis of Ca-based sorbents able to provide superior reactivity [8,44,46]. In this framework, the utilization of fine CaO particles has been recently proposed as a promising alternative solution [7,47]. In fact, it has been demonstrated that the sintering and pore-plugging phenomena can be remarkably hindered if CaO small particles are employed, thus improving the obtainable multicyclic performances [7,47]. Besides this, it has been reported that the use of fine CaO particles, thanks to their intrinsic high surface to volume ratio, makes it possible to enhance the gas–solid contact efficiency with respect to coarser CaO particles [37,48]. Likewise, the use, in small concentrations, of inert nano-sized particles as additives to fine cohesive limestone powders has been also proved to be a viable option to improve the flowability and reactivity performances of the system [49,50].

Therefore, in light of all the possible applications reviewed above, it is evident that it is necessary to develop new technologies to process these particles in big amounts. In this framework, several techniques can be used to handle and disperse fine/ultrafine powders in a continuous manner. Among these, fluidization is one of the most effective technological options. Indeed, fluidized beds can rely on different advantages, such as: high gas–solid contact area and efficiency, large heat/mass transfer coefficients, relatively easy control of the bed temperature uniformity, high particle flowability, flexibility in terms of types of powders to be processed, and suitability for large-scale applications [51].

However, regardless of these benefits of fluidized beds in handling powdered materials, it must be considered that fine/ultrafine particles, i.e., belonging to Geldart’s C group, cannot be fluidized under ordinary fluidization conditions due to their intrinsic cohesive nature [1,2,52]. Indeed, these particles are characterized by strong interparticle forces (IPFs), causing the natural formation of large aggregates, due to the particles tendency to stick to one another giving rise to larger structures as large as hundreds of microns [1,2,53]. This agglomeration tendency causes, in turn, channeling/plugging phenomena, thus hindering the possibility to obtain a proper fluidization quality under ordinary conditions [2,53,54,55,56].

Therefore, fine/ultrafine powders can be fluidized only with the use of some kind of externally assisted fluidization devices, namely it is necessary to introduce some kind of external energy in order to overcome the strong inter-particle forces. Among the different alternatives (magnetic [57,58], electric fields [59,60], mechanical vibrations [61,62], etc.), sound-assisted fluidization has been proposed as one of the most effective solutions [2,55,63]. In particular, it has been reported that agglomeration, channeling and/or slugging phenomena can be efficiently hindered if a suitable acoustic field, i.e., capable of overcoming the IPFs, is applied, thus assuring the possibility to achieve a proper fluidization regime [19,37,64,65].

In this context, the sound-assisted fluidized bed reactor (SAFB) designed and set-up in Naples by our research group has proven itself as a consistent device for the fundamental and phenomenological study of the fluidization behavior of the fine/ultrafine cohesive powders at atmospheric pressure [2,54,55,66,67]. More recently, the SAFB has also been upgraded to be used for both low- and high-temperature chemical processes, such as CO_2_ adsorption and Ca-looping, studied in the framework of both CCS and TCES-CSP applications [3,4,5,6,15,19,20,21,36,37,64,65,68,69].

In this work, the main results recently obtained by means of the SAFB setup are reviewed, particularly focusing on how the application of the acoustic perturbation reflects not only on the fluid dynamics of the system, but also on the performances/outcomes of the specific chemical processes carried out therein.

## 2. Experimental Set-Up and Procedure

### 2.1. SAFB

**Figure 1:**
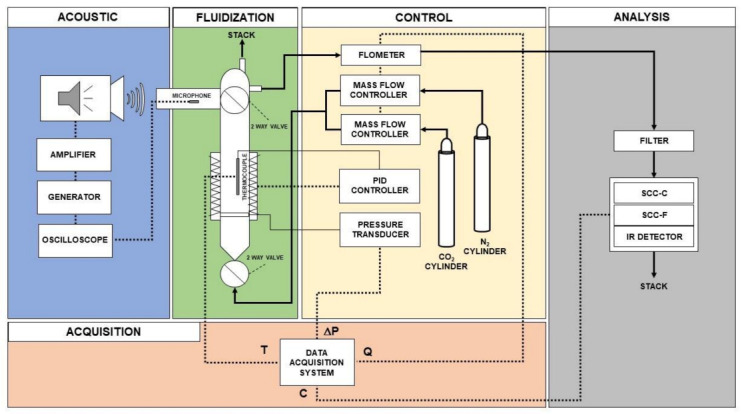
Sound-assisted fluidized bed reactor (SAFB): scheme of the experimental set-up.

Figure 1 reports the scheme of the SAFB designed and set-up in Naples by our research group and used in all the works reviewed in the present manuscript. It is based on the initial setup originally conceived by Chirone et al. [70,71] to study the fundamental behavior of cohesive powders at atmospheric pressure. The SAFB mainly consists of five parts: (i) the fluidization/reaction section; (ii) the acoustic section; (iii) the control section; (iv) the analysis section; and (v) acquisition section.

The fluidization section is made of a fluidization column in Plexiglas, Pyrex, or quartz, depending on the operating temperature. The column is provided with a gas distributor, consisting of a quartz porous plate. The section situated below the gas distributor is a wind-box. In particular, the proper gas distribution below the quartz plate is assured by a quartz rings filling. A pressure probe (linked to pressure transducers) located just above the gas distributor makes it possible to directly measure the pressure drops across the bed, i.e., without the contribution of the distributor plate.

The acoustic section consists of a sound-generation/propagation system. In particular, the acoustic field is generated by a digital signal generator, which is capable of generating an electrical sine wave of specified frequency (f). Then, the signal, amplified in a power audio amplifier rated up to 400 W, is sent to an 8 W woofer loudspeaker and finally introduced in the fluidization column through a specifically designed sound wave guide, which is linked to the upper part of the fluidization column so that the sound wave propagates from the top to the bottom of the column. In particular, the geometry of this sound wave guide and, in turn, the reciprocal position of the loudspeaker and the fluidization column, have been properly conceived in order to protect the loudspeaker from any possible elutriated materials and/or from the heat emitted from the bed in the case of high temperature tests. The response of the bed to the acoustic perturbation is monitored through a microphone located in the upper part of the fluidization column and linked to an oscilloscope. This makes it possible to monitor and adjust the intensity (SPL) of the acoustic field according to the signal received from the bed through the microphone. Considering that the intensity of the acoustic fields generated in our research activity is relatively high (>110 dB) [3,54], the experimental set up envisages a noise insulation system based on the principles of the Helmholtz resonator [72,73,74]. The resonator simply consists of a cavity connected to the system of interest through one or several short and narrow tubes (necks). The resonator has a characteristic resonance frequency (to which corresponds the maximum oscillation), which is strictly dependent on its geometry, i.e., the volume of the cavity and the length of the neck [72,73,74]. Therefore, by properly selecting these geometrical parameters, it has been possible to make the resonance frequency characteristic of the resonator fall inside the frequency domain of interest, thus effectively dampening the noise [72,73,74].

The control section consists of all the devices used to set, measure, and control the operating variables of the experiments, i.e., the inlet gas flow rates and temperature. A set of mass flow meters and controllers (Brooks) with different full-scale values (ranging from 1 to 300 NL h^−1^) is used to set and control the inlet gas flow rate. Likewise, a mass flowmeter (Brooks) located at the exit of the column is used to measure the flow rate of outlet stream. The gas feed is prepared using separate high purity cylinders (99.999%) containing the gaseous species (N_2_ and CO_2_) required for the experiments. In regards to the temperature, it is set, measured, and controlled by a PID controller equipped with a type K thermocouple (positioned at the center of the bed), which is linked to an ad-hoc designed electric heating jacket (Tyco Thermal Controls GmbH) specifically designed with an open window in order to visually assess the fluidization quality.

The analysis system is made of a continuous online analyzer (ABB, AO2020) with an infrared (URAS 14) detector (with a 0–100 CO_2_ vol.% full range) able to measure the outlet CO_2_ concentration continuously. In particular, a part of outlet stream is sampled by means of a sample gas feed unit (ABB, SCC-F) and sent to the analyzer, whereas the residual part is sent to the stack. The system is also equipped with a Peltier cooler (ABB, SCC-C) for cooling the sample and separating and removing any possible condensate. Besides that, the analysis system is also protected from any possible elutriated particles by a system of filters placed downstream from the exit of the fluidization column. With reference to the elutriation phenomena, it should be considered that under the application of acoustic fields, the elutriation of fine particles from the fluidized bed is remarkably reduced, thus limiting possible related issues such as clogging of valves [2].

Finally, in regards to the data acquisition system, the pressure drops (ΔP), temperature (T), gas flowrate (Q), and concentration (C) signals are continuously recorded and elaborated on a PC, at a sampling rate of 1 Hz, via LabView software.

### 2.2. Materials

All the materials tested in the SAFB are reported, with their main properties, in Table 1. Clearly, except for the silica sand, belonging to the Geldart’s A group, all the powders are characterized by dimensions lower than 30 μm, i.e., they all belong to the Geldart’s C group. However, they can be categorized in two main groups on the basis of their primary particle size: nano-sized and micro-sized powders.

In particular, the nano-powders are: Al_2_O_3_, Fe_2_O_3_, ZrO_2_, and CuO, all of them produced by Sigma-Aldrich. Whereas, in regards to the micro-powders, the two commercial activated carbons are an activated carbon DARCO FGD (Norit), AC Norit, and an activated charcoal powder (Sigma Aldrich, Milan, Italy), AC Sigma. The metal organic framework (MOF), HKUST-1, has been synthesized according to the procedure reported in [68]. The natural limestone, CaCO_3_, comes from Belchite quarries (Spain) and has been supplied by OMYA. Finally, the synthetic CaO is produced by Sigma–Aldrich.

### 2.3. Fluidization Tests

All the fluidization experiments have been carried out in the SAFB at atmospheric pressure and at temperatures ranging from 25 to 850 °C under ordinary ad sound-assisted operating conditions. The fluidizing gas is N_2_ in order to avoid air moisture that could intensify the powder cohesiveness. In regards to the sound-assisted tests, acoustic fields with different sound frequency (f: 20–300 Hz) and intensity (SPL: 120–150 dB) have been applied in order to study the effect of the acoustic parameters on the fluidization behavior.

Each fluidization experiment consists in measuring and recording the pressure drops as a function of the superficial gas velocity (u), i.e., by both decreasing (DOWN) and increasing (UP) u. All of the results shown in this work have been obtained from the DOWN curves. Then, the main fluidization parameters are evaluated by working out to the obtained experimental data, i.e., the minimum fluidization velocity (u_mf_) and the average aggregate size (d_agg_).

In regards to u_mf_, the well-known graphical procedure is applied to the experimental pressure drop curve: u_mf_ is evaluated by crossing the line that fits the data for flow through a packed bed and the horizontal line that fits the data for the fully fluidized bed [75].

In regards to d_agg_, the correlation proposed by Wen and Yu [75] has been applied using the experimental values of u_mf_. In particular, an apparent density, smaller than the primary particle density, has been considered for the aggregates [54].

### 2.4. CO_2_ Adsorption Tests

Dynamic adsorption/desorption experiments have been carried out in the SAFB under atmospheric pressure and different adsorption/desorption temperatures (T_ads_, T_des_) ranging from 25 to 150 °C and either with or without the assistance of the sound perturbation. In particular, relatively low values of CO_2_ partial pressures (P_CO_2__: 0.05–0.20 atm) have been tested in order to simulate the composition of a typical post-combustion flue gas. The feeding stream in both the adsorption and desorption step is set so that superficial gas velocity is sufficiently bigger than the u_mf_ of the tested sorbent, thus assuring that the whole bed is fully fluidized.

In particular, cyclic tests of CO_2_ capture and recovery have been performed according to the TSA approach. Therefore, each cycle consists of an adsorption step, in which the CO_2_ is captured on the solid sorbent, and a subsequent desorption step, in which the sorbent is regenerated by increasing the temperature of the system and the CO_2_, previously captured, is released.

Adsorption: Before the adsorption test, a preliminary step is always performed: the sorbent is firstly dried/cleaned by flowing N_2_ at 150 °C and atmospheric pressure for 60 min (this is necessary to remove any trace of water, which would end up reducing the CO_2_ adsorption capacity). Then, it is pre-conditioned by fluxing N_2_ through the system until the system reaches the desired temperature. After this preliminary stage, the adsorption step is started, in which the feed to the column is switched to a CO_2_/N_2_ gas mixture of specified composition (C_0_). Thanks to the continuous monitoring of the CO_2_ concentration in the outlet stream, the breakthrough (BT) curves, consisting in the plot of C/C_0_ versus time, are obtained. Then, the BT curves are elaborated in order to calculate some important adsorption parameters:

- q_e_—the amount of CO_2_ adsorbed per unit mass of adsorbent at the thermodynamic equilibrium. In particular, this is evaluated from the integration of the BT curve according to the following mass balance:
  (2) $$q_{e}=\frac{1}{m}\int_{0}^{t_{s}}\left(F_{{\mathrm{CO}}_{2},\mathrm{in}}-F_{{\mathrm{CO}}_{2},\mathrm{out}}\right)\mathrm{dt}$$
  where m is the sorbent mass, F_CO_2___,in_ and F_CO_2___,out_ are the inlet and outlet CO_2_ molar flowrate, respectively, and t_s_ is the saturation time;
- t_b_—the BT time, namely, the time in which the outlet CO_2_ concentration is the 5% of the feed concentration. Technically, in real industrial adsorption processes, it is the time at which the adsorber is taken off-line for regenerating the sorbent, which means that larger values of t_b_ correspond to the more effective capture capacities;
- Δτ—a time parameter providing information on the kinetics of the adsorption process. The smaller Δτ is, the faster the adsorption will be. In particular, it is representative of the slope of the linear part of the BT curve and it is evaluated as t_95_—t_b_ (with t_95_ being the time for which the outlet CO_2_ concentration reach 95% of the concentration in the feed), which means that higher values of Δτ represents steeper BT curves;
- ψ—the fraction of bed used at BT, namely the percentage of the total captured CO_2_ (q_e_) which is adsorbed at the BT time, t_b_.

Desorption: In regards to the desorption step, different regeneration modes have been used:

- non-isothermal purge—in this mode of operation, the heating happens contextually to the N_2_ purging step. In particular, once the adsorption step is concluded, the column is heated up (20 °C/min) to the selected T_des_ and, at the same time, the feed (Q_IN_) is switched from the CO_2_/N_2_ mixture, used during the previous adsorption step, to pure N_2_ (Q_p_) to purge the bed.
- isothermal purge—in this mode of operation, the purging step happens isothermally. In particular, once the adsorption step is concluded, the column is isolated so that all the desorbed CO_2_ is kept inside the column. To do so, the inlet and outlet of the column are closed by means of two-way valves and, in the case of sound-assisted adsorption tests, the acoustic field is switched off and the system is heated up to the selected desorption temperature. Then, once the set T_des_ is reached, the purging step is started, namely, the acoustic field is switched on, in the case of sound-assisted tests, the feed to the column is opened fluxing N_2_ and the column exit is un-sealed. In this way, the CO_2_ already desorbed from the adsorbent, which has been trapped inside the isolated column, and the CO_2_ still adsorbed on the sorbent (i.e., the CO_2_ that can be released only by reducing the CO_2_ partial pressure) exits from the column diluted in the N_2_ stream.

In analogy to the adsorption step, thanks to the continuous monitoring of the CO_2_ concentration in the outlet stream, the desorption peaks are elaborated in order to calculate some important desorption parameters:

- R—the CO_2_ recovery level, defined as the percentage of the previously captured CO_2_ which is recovered during the desorption step;
- t_d_—the time needed for CO_2_ desorption at a fixed value of R;
- C_m_—CO_2_ purity, expressed as the average CO_2_ concentration in the desorbed stream.

### 2.5. Ca-Looping Tests

CaL tests have been performed for both CCS and TCES applications either with or without the assistance of the acoustic perturbation. However, only the tests for TCES have been performed in the SAFB in Naples, whereas those for CCS have been performed in the experimental apparatus set up in Seville in collaboration with the University of Seville. Detailed information on this experimental apparatus can be found in [37].

The experimental procedures used for both the CCS and TCES are similar, they only differ for the operating temperatures of the carbonation and calcination steps. In fact, the optimal operating conditions to perform the CaL process are strictly dependent on the particular application. In the case of CCS applications: the calcination, whose conditions are dictated by the need of extracting from the calciner high-concentration CO_2_ stream to be compressed and sequestered, is performed at high temperature (∼950 °C) under high CO_2_ partial pressure. The carbonation, whose conditions are dictated by the fixed concentration of the combustion flue gases, is performed under low CO_2_ partial pressure (∼0.15 bar) at the lowest possible temperature (∼650 °C) able to favor the reaction thermodynamically [5]. In the case of TCES applications: since there is no more CO_2_ capture/storage issue, relatively low temperatures (~750 °C) and CO_2_ partial pressures (i.e., by using a gas easily separable from CO_2_, such as superheated steam or helium) are employed to perform the calcination. On the contrary, aiming at achieving high global efficiency for energy storage and electricity generation, high CO_2_ partial pressures and temperatures (around or above 800 °C) are employed to perform the carbonation [5].

In both cases, CCS and TCES tests, the sorbent is subjected to a sound-assisted (150 dB–120 Hz) pre-treatment step, in which it is pre-calcined in N_2_ at 900 °C in order to obtain a completely de-carbonated sorbent for the following carbonation step.

CCS: After the pretreatment, the sorbent is subjected to a carbonation step, performed at 650 °C feeding a mixture of 15% CO_2_/85% N_2_ *v/v* until the saturation of the sorbent. Then, a calcination step is performed at 900 °C in N_2_ atmosphere to fully de-carbonate the sorbent.

TCES: The operating conditions have been carefully chosen in order to simulate a CaL–CSP integration scheme in which the calcination is carried out at low temperature feeding helium and the carbonator is integrated with a closed CO_2_ Brayton cycle [76]. In particular, after the pre-treatment, the sorbent is subjected to a carbonation step carried out at 850 °C using a mixture of 70% CO_2_/30% N_2_ *v/v* until the sorbent is saturated. Then, a calcination step is performed at 750 °C in N_2_ atmosphere until the sorbent is completely de-carbonated.

Also for these tests, the outlet CO_2_ concentration profiles are elaborated in order to calculate the carbonation conversion, X.

## 3. Results

In the following paragraphs, the main results obtained by means of the SAFB setup are presented and reviewed, pointing out how the application of the acoustic perturbation affects the fluid dynamics of the system and, in turn, also the performances/outcomes of the specific chemical processes carried out therein.

### 3.1. Fluid Dynamics of Sound-Assisted Fluidized Beds

**Figure 2:**
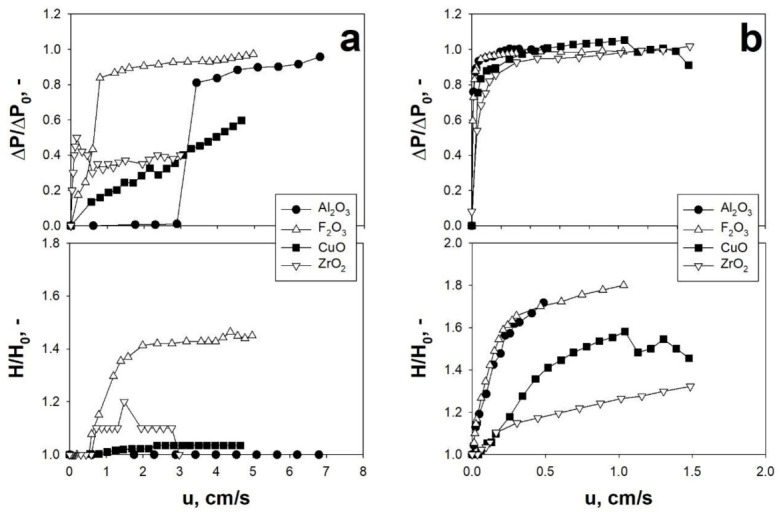
Dimensionless pressure drop (ΔP/ΔP_0_) and bed expansion (H/H_0_) curves obtained for different nano-sized particles: (**a**) ordinary tests; (**b**) sound-assisted tests (140 dB–120 Hz). T = ambient temperature. Adapted from [55] with permission.

**Figure 3:**
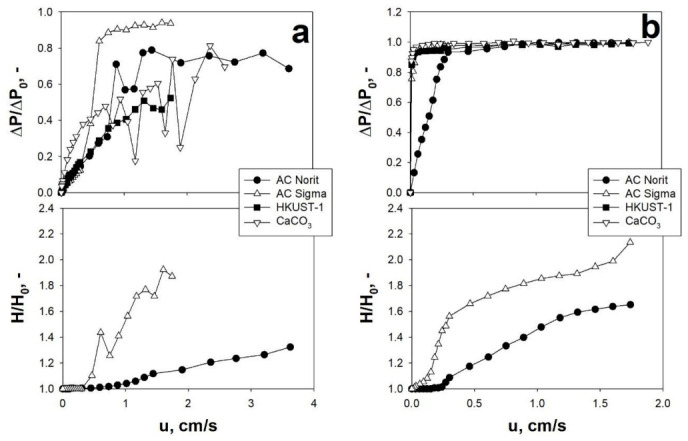
Dimensionless pressure drop (ΔP/ΔP_0_) and bed expansion (H/H_0_) curves obtained for different micro-sized particles: (**a**) ordinary tests; (**b**) sound-assisted tests (AC Norti/AC Sigma/HUST-1: 140 dB–80 Hz; CaCO_3_: 150 dB–120 Hz). T = ambient temperature. Adapted from [3,5] with permission.

Studying the behavior of different fine/ultrafine cohesive particles, it has been shown that, regardless of the specific particle size, the application of an acoustic perturbation, with proper f and SPL, makes it possible to either achieve an appropriate fluidization regime or improve their fluidization quality [2,55], in terms of ideal-like pressure drop curves and affecting the values of all the most important fluidization parameters (lower values of u_mf_ and d_agg_). In this framework, Figure 2 and Figure 3 report, as examples, the fluidization and expansion curves obtained for different nano-sized and micro-sized powder, respectively, under ordinary and sound-assisted conditions.

Clearly, from the analysis of Figure 2a and Figure 3a, it is evident that when the powders are fluidized without any external excitation, the fluidization quality is relatively poor, as confirmed by the dimensional pressure drops being unstable and lower than 1, which means that a fraction of the bed remains not fluidized. On the contrary, when the acoustic perturbation is applied, the fluidization curves become more regular, with the whole bed fluidized (ΔP/ΔP_0_ always reaching 1) and higher values of expansion ratio (Figure 2b and Figure 3b). These experimental evidences are due to the fact that the application of a proper sound excitation is able to overcome the strong interparticle forces, thus hindering the agglomeration, channeling, and/or slugging phenomena. More specifically, the application of the acoustic perturbation generates both inertia and viscous forces that induce an oscillatory motion of gas molecules and solid particle/aggregates. In particular, depending on the dimension of the fluidizing aggregates (i.e., cluster and sub-clusters), the entity of this motion can be more or less intense [53]. This results in a relative motion between particle aggregates of different dimensions, which, in turn, induces a dynamic break-up mechanism of the bigger clusters into smaller and more easily fluidizable sub-clusters, as theoretically explained in [54].

**Figure 4:**
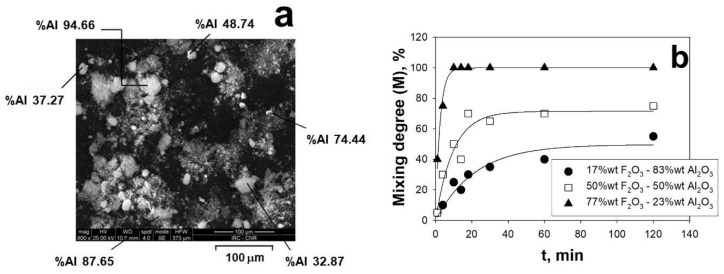
(**a**) SEM images and EDS analysis of the fluidizing aggregates taken from the bed after 14 min during the sound-assisted mixing test (17 wt.% F_2_O_3_–83 wt.% Al_2_O_3_); (**b**) time evolution of the aggregates mixing degree for different relative amounts of the two nano-powders. T = ambient temperature. Adapted from [66] with permission.

The dynamics of the break-up mechanism has been also clearly highlighted investigating the mixing of different nano-sized powders (F_2_O_3_ and Al_2_O_3_). In particular, combining the color tracing method with SEM/EDS morphological/chemical analysis of samples taken from the bed at different times, it has been demonstrated that the acoustic perturbation promotes a dynamic evolution of the fluidizing aggregates. In fact, the fluidizing aggregates, initially formed by only one of the two powders, are not static but dynamic structures, as confirmed by the fact that their chemical composition varies with time during the sound-assisted mixing test. Due to the aggregates continuously breaking-up and re-aggregating, there is an actual exchange of material at the micron scale which creates hybrid aggregates containing both of the two types of particles. More specifically, while the bed appears macroscopically well mixed after just few minutes, the local mixing inside the aggregates takes longer times. These considerations are clearly evidenced in Figure 4a,b reporting, respectively, the chemical composition of the hybrids aggregates and the time evolution of the mixing degree M, where M(t) is the ratio between the number of aggregates having a chemical composition different from the theoretical value of less than 10% and the total number of aggregates analyzed at the time t [66].

#### 3.1.1. Effect of Sound Intensity and Frequency

**Figure 5:**
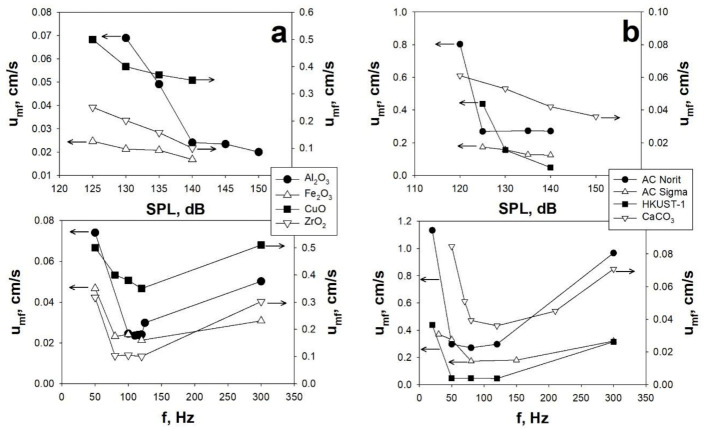
u_mf_ as a function of SPL, at fixed frequency, and frequency, at fixed SPL, for: (**a**) nano-sized particles (fixed f = 120 Hz; fixed SPL = 140 dB); (**b**) micro-sized particles (AC Norti/AC Sigma/HUST-1: fixed f = 80 Hz, fixed SPL = 140 dB; CaCO_3_: fixed f = 120 Hz, fixed SPL = 150 dB). T = ambient temperature. Adapted from [3,5,55] with permission.

Aiming at highlighting the effect of the acoustic parameters, i.e., SPL and f, on the fluidization behavior of fine/ultrafine particles, tests have been performed in the SAFB applying acoustic fields of different SPL (120–150 dB) and f (20–300 Hz). In this framework, Figure 5a,b report the effect of SPL and f on the fluidization behavior, in terms of u_mf_, of different nano-sized and micro-sized particles, respectively.

Clearly, in regards to the effect of SPL, it has been found that increasing intensities of the acoustic perturbation lead to a general enhancement of the fluidization quality (Figure 5). Indeed, the strength of the acoustic field is proportional to SPL [2,77] and, as a consequence, a minimum acoustic energy (SPL > 120 dB) is required to start the break-up mechanism, which, in turn, promotes the fluidization process. Besides that, an increase of SPL results in enhancing the fluidization quality, as clearly confirmed by u_mf_ decreasing with increasing SPL (Figure 5). Indeed, larger values of SPLs are representative of larger amount of external energy introduced inside the system, which means that the break-up mechanism is more effective, i.e., the larger fluidizing aggregates are disrupted in smaller and more fluidizable structures. However, it must be also considered that there is some experimental evidence that at very high values of SPL, this positive effect may be reversed because of the collision between particles and/or agglomerates becoming more and more probable, thus leading to an increase of the aggregate size [78].

On the contrary, in regards to the effect of sound frequency on the fluidization quality, it has been found that there is a non-monotonic correlation, indeed, it can be found an optimum range of frequency (80–120 Hz) in the proximity of to the natural frequency of the particle bed. This is clearly shown in Figure 5, where the existence of the optimum range of frequency, providing the lowest values of u_mf_ (i.e., more efficient break-up mechanism), is evident for all the tested nano-sized and micro-sized powders. In regards to the explanation of this experimental evidence, it must be considered that for too small values of sound frequency, there is no relative motion between smaller and larger aggregates and, as a consequence, there is no aggregate break-up [2,53,54]. On the contrary, for too high, values of sound frequency, the propagation of the acoustic waves though the particulate bed cannot take place properly [2,53,54]. In fact, there is a proportional correlation between the sound absorption coefficient and the square of sound frequency [2,53,54]. Therefore, when the sound frequency is too high, the upper part of the bed absorbs most of the sound energy, whereas, the bottom of the bed is only interested by a weakened sound energy, thus meaning that the large aggregates located there are not efficiently disrupted [2,53,54].

#### 3.1.2. Effect of Temperature

It is generally accepted that the influence of the temperature on the fluidizing gas characteristics (density and viscosity) can be used to explain how the fluidization behavior is affected by the temperature only if the fluidization is controlled by the hydrodynamic forces (HDFs) [2,53,54,79,80,81]. On the contrary, other aspects must be taken into account if the IPFs are either comparable with or predominate over the HDFs, as typical in the fluidization of fine/ultrafine particles [53,54]. Indeed, the temperature affects as the gaseous phase, in terms of density and viscosity variations, as the solid phase, in terms of intensification of IPFs [53,54]. In this context, several authors studied the effect of the temperature on the fluidization behavior of different types of powders, reporting an increase of the IPFs with increasing values of the temperatures [2,53,54,79,80]. It has been also found that, in spite of the fluidization of coarser powders being generally controlled by HDFs and not affected by IPFS, non-cohesive A powders can switch to C-type fluidization behavior when the temperature is increased, as a consequence of the intensified IPFs outbalancing the HDFs [81].

**Figure 6:**
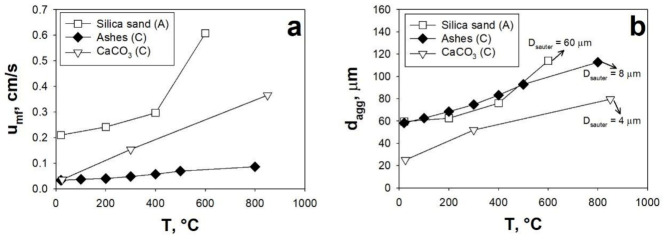
(**a**) u_mf_ as a function of the temperature; (**b**) d_agg_ as a function of the temperature. SPL = 150 dB; f = 120 Hz; A = powder belonging to the A group of Geldart’s classification; C = powder belonging to the C group of Geldart’s classification. Adapted from [5,54] with permission.

The effect of the temperature on the fluidization behavior of A and C powders has been also investigated in the SAFB. According to this general consensus of the works available in the literature, it has been found that increasing temperatures negatively affect the fluidization quality due to the intensification of IPFs. Indeed, regardless of the powders being either A or C type, the fluidization parameters, u_mf_ and d_agg_, are increased when the temperature is increased, as clearly shown in Figure 6. This can be explained considering that, as consequence of the intensified IPFs, the achievement of a fluidization regime becomes more and more difficult due to a less effective break-up mechanism. Indeed, with the intensification of IPFs the cohesive character of the powder is enhanced, i.e., with increasing temperatures, more and more particles adhere to one another to form larger aggregates (higher values of d_agg_) (Figure 6b) which lead to larger values of u_mf_ (Figure 6a).

In particular, in regards to the C type powders, it is clear that the efficiency of the acoustic perturbation decreases as the temperature is increased. In fact, as clearly inferable from Figure 6b, the fluidizing aggregate size, which is always larger than the powder average size (Ashes = 8 μm, CaO = 4 μm—Table 1), increases more and more as the temperature is increased, becoming 13–15 times larger than the Sauter diameter around 800 °C.

Likewise, also in the case of the A type powder, increasing temperatures lead to a reduced efficiency of the acoustic field in counteracting the effect of IPFs. In fact, even though the fluidizing parameter increase whether or not the acoustic field is applied, the acoustic field keeps its effectiveness up to 200 °C. In fact, the fluidizing aggregates are broken down to the Sauter diameter (60 μm—Table 1) only for temperatures smaller than 200 °C, as confirmation of an effective break-up mechanism. On the contrary, when the temperature is increased beyond 200 °C, the effectiveness of the acoustic field decrease until becoming negligible at 600 °C, where no difference is observed between the ordinary and sound-assisted tests (d_agg_ is about two times the D_sauter_ of the powder) (Figure 6b).

### 3.2. Temperature Swing Adsorption (TSA)

Since fine/ultrafine powders have great potential to be used for the CO_2_ adsorption process, the SAFB has been proposed in a TSA configuration (TSA-SAFB) as possible technology for the handling and processing of these materials in large quantities. In particular, an intense research activity has been performed to highlight and demonstrate that fine/ultrafine powdered sorbents, regardless of their specific chemical nature, can be directly used in the TSA-SAFB, i.e., they can be used as free-flowing powders, without being previously treated. In particular, there is no need to perform a previous pelletization step (which, on the contrary, would be necessary to face the huge pressure drops of fixed bed reactors of fine particles), which ends up in decreasing the sorbent surface area and, consequently, its adsorption capacity for CO_2_, especially for some kinds of novel cutting-edge sorbent materials [82]. Besides this crucial benefit, the TSA-SAFB has been also proved to significantly improve the CO_2_ adsorption and desorption performances of fine/ultrafine powdered sorbents with respect to their use in other standard reactor configurations (e.g., ordinary fluidized beds and fixed beds).

#### 3.2.1. Adsorption

**Figure 7:**
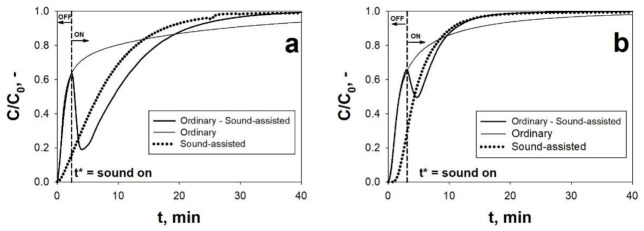
(**a**) CO_2_ breakthrough (BT) curves obtained for AC Norit during an ordinary fluidization test, a sound-assisted fluidization test and during a test in which the sound is switched on at t = t^*^. C_0_ = 10 vol.%; u = 1.5 cm/s; SPL = 140 dB; f = 80 Hz; T = ambient temperature. (**b**) CO_2_ BT curves obtained for HKUST-1 during an ordinary fluidization test, a sound-assisted fluidization test and during a test in which the sound is switched on at t = t*. C_0_ = 10 vol.%; u = 1.5 cm/s; SPL = 140 dB; f = 120 Hz; T_ads_ = ambient temperature. Adapted from [37,68] with permission.

In regards to the adsorption step, Figure 7 reports the BT curves obtained for two adsorbents belonging to two different class of adsorbent materials, i.e., activated carbons and MOFs, under ordinary and sound-assisted fluidization conditions. Table 2 reports the adsorption parameters calculated from the elaboration of the BT curves.

The analysis of Figure 7 and Table 2 shows that the application of the acoustic perturbation remarkably improves the CO_2_ adsorption performances of both the two sorbents. Indeed, all the adsorption parameters of AC Norit and HKUST-1 are enhanced in the sound-assisted test with respect to the ordinary test: (i) the equilibrium adsorption capacity, q_e_, is increased of about 20% and 45%; (ii) the BT time, t_b_, is increased of about 5 times; and (iii) the fraction of bed utilized at t_b_, ψ, is increased of about 4 and 3 times for AC Norit and HKUST-1, respectively. Besides this, it is also evident that the adsorption kinetics is also enhanced when the system is assisted by the acoustic excitation, as clearly evidenced by the BT curves becoming steeper and shorter. Indeed, the saturation time, t_s_, is roughly reduced to a half and Δτ is reduced of about 60%, thus meaning that the CO_2_ adsorption is speeded up when the acoustic perturbation is applied.

These experimental evidences can be explained considering that without the assistance of the acoustic perturbation, i.e., under ordinary conditions, the fluidization quality is extremely poor and characterized by strong instability. This is clearly evidenced by the fluidization curve obtained for both the sorbents (Figure 3a). Due to cohesive nature of the sorbents, the gas–solid contact efficiency is dramatically hindered and, therefore, most of the reactive gas (CO_2_) bypasses the bed through the formation of channels rather than actually permeating the bed. Likewise, from the point of view of the solid phase, a great amount of the sorbent does not actually take part in the adsorption process, which means that a great amount of adsorbent surface is not exploited, being completely precluded to the gaseous phase. Then, once the most readily available adsorption sites (i.e., those located on the surface of the channels) are quickly saturated, the outlet CO_2_ concentration increases, due to the above-mentioned by-pass phenomenon, with a sudden increase of the slope of the BT curve. Then, after this sharp rise, the slope of the BT curve abruptly decreases approaching the saturation with a very slow tail of the BT curve, which accounts for most of the adsorption process. In particular, the saturation of the bed occurs very slowly due to the fact that only a small fraction of the solid and gaseous reactants takes part in the adsorption process.

On the contrary, when the acoustic perturbation is applied to the system, the BT curves show a more regular shape, i.e., with no sudden initial increase and slow tail, thanks to the enhanced of the fluidization quality and, in turn, of the gas–solid contact efficiency. More specifically, the general improvement of the CO_2_ adsorption performances observed during the sound-assisted tests is due to the characteristic dynamic break-up and re-aggregation mechanism of the fluidizing aggregates, as distinctive feature of the sound-assisted fluidization process. In fact, this mechanism leads to the continuous renewing of the sorbent particles exposed to the fluid phase and, in turn, of the active sites available to the CO_2_ adsorption process.

This strong connection between the CO_2_ adsorption process and the sound-assisted fluidization process has been clearly evidenced by performing an OFF–ON test, in which the acoustic field is switched on only at a time t = t^*^, i.e., soon after the above-discussed change of slope characteristic of the ordinary tests, (Figure 7). It is evident that when the acoustic perturbation is switched on, at t = t*, the CO_2_ concentration profile suddenly decreases and, after few minutes, restart the rising with a sound-assisted-like shape. This experimental evidence is the confirmation that the acoustic perturbation makes it possible to better exploit the sorbent surface. In fact, the sorbent surface, that is precluded to the gaseous phase while the sound is switched off in the first part of the test, suddenly becomes available, as soon as the acoustic field is switched on. As a consequence, the CO_2_ concentration abruptly drops down, due to the renewed sorbent adsorption capacity.

**Figure 8:**
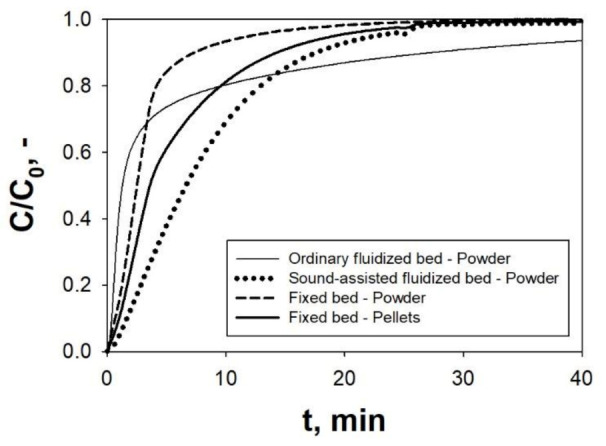
BT curves obtained under ordinary fluidization, sound-assisted fluidization and fixed bed conditions with AC Norit in powdered and pelletized form. u = 1.5 cm/s; C_0_ = 10 vol.%; SPL = 140 dB; f = 120 Hz. T_ads_ = ambient temperature. Adapted from [37] with permission.

The superiority of the SAFB reactor configuration to perform the CO_2_ adsorption process on fine/ultrafine sorbents have been also proved by testing the same sorbent (AC Norit) in a fixed bed reactor. In particular, the fixed bed experiments have been performed with the sorbent in both powdered and pelletized (pellet size: 180–400 μm) form. The comparison of the obtained BT curves is reported in Figure 8. It is evident, in regards to the fixed bed tests, that the pelletization improves the performances with respect to the free-flowing powders. This is due to the fact of the strong cohesive nature of the fine sorbent, the gas flows through the bed through channels rather than permeating it. However, whether pelletized or not, when used in a standard fixed bed reactor, the sorbent provides remarkably worse CO_2_ adsorption performances than when used in the SAFB. In particular, in the SAFB, its adsorption capacity can be increased of about 76% with respect to the fixed bed operation with the pellets [4]. This is most likely due to diffusive resistances inside the pellets, hindering the full exploitation of the sorbent surface.

**Figure 9:**
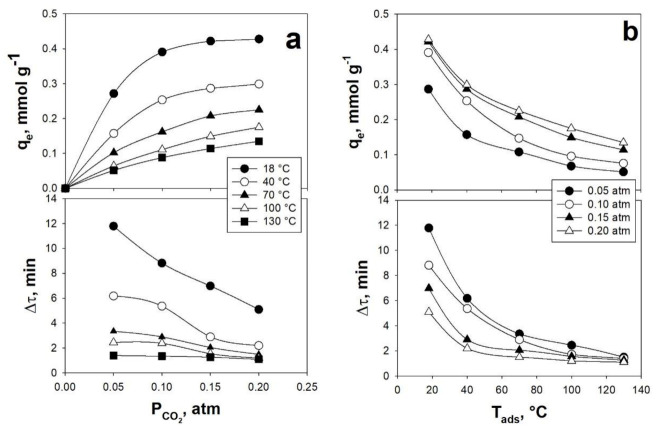
q_e_ and Δτ obtained for AC Norit as functions of: (**a**) P_CO_2__ at different values of T_ads_ and (**b**) T_ads_ at different values of P_CO_2__. u = 1.5 cm/s. Adapted from [69] with permission.

The effect of some important operating variables on the CO_2_ adsorption process, i.e., CO_2_ partial pressure, P_CO_2__, and temperature, T_ads_, has also been investigated in the SAFB (Figure 9).

It is clear, from the analysis of Figure 9, that the pressure and the temperature have an effect on both the CO_2_ adsorption thermodynamics and kinetics.

In regards to the CO_2_ adsorption thermodynamics, it is positively affected by increasing values of P_CO_2__, whereas, it is negatively affected by increasing values of T_ads_. Indeed, higher values of P_CO_2__ lead to higher values of q_e_, due to the fact that the CO_2_ adsorption process is thermodynamically controlled by P_CO_2__ [17]. On the contrary, q_e_ decreases with increasing T_ads_, due to the adsorption being an exothermic process [11].

In regards to the CO_2_ adsorption kinetics, it is positively affected by increasing both P_CO_2__ and T_ads_. In fact, higher values of P_CO_2__ lead to lower values of Δt, i.e., faster adsorption processes, due to the fact that longer times are needed by the CO_2_ concentration to go through the bed [11,16]. Likewise, larger values of T_ads_ also result in smaller values of Δt, due to the enhancement of the mass-transfer coefficients at higher temperatures [11,16].

#### 3.2.2. Desorption

**Figure 10:**
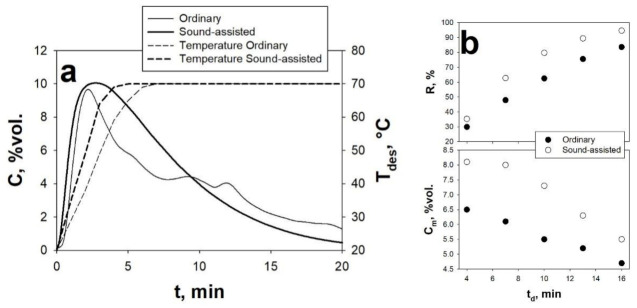
(**a**) Desorption peaks of the AC Norit obtained, using the non-isothermal purging mode, under ordinary and sound-assisted conditions. The sorbent temperature profile is also reported (dashed lines). C_0_ = 10 vol.%. (**b**) R and C_m_ as functions of t_d_. Q_p_ = 67.8 NL h^−1^; heating rate = 20 °C/min up to T_des_ = 70 °C. Adsorption step: ordinary fluidization; Q_IN_ = 67.8 NL h^−1^; C_0_ = 10 vol.% Adapted from [20] with permission.

In regards to the desorption step, Figure 10a reports the desorption peaks and temperature profiles inside the bed obtained for the AC Norit under ordinary and sound-assisted fluidization conditions, using the non-isothermal purging mode described in the experimental section.

The analysis of Figure 10 evidences that, under both ordinary and sound-assisted fluidization conditions, the CO_2_ desorption profile shows the typical shape of a peak followed by a final tail, which is indicative of the CO_2_ desorption occurring slowly in the final stages due to the reduction of the driving force. Also, it is important to underline that, regardless of the fact that the process is carried out under sound-assisted or ordinary conditions, the sorbent is always fully regenerated, i.e., the desorbed CO_2_ is always the same as the CO_2_ previously adsorbed. However, even though the shapes of the ordinary and sound-assisted profile are similar, it is evident, in analogy to the results obtained for adsorption step, that the desorption profile is smoother and more regular under sound-assisted fluidization conditions. When no acoustic field is applied, on the contrary, a clear instability can be evidenced, which is caused by the fact that the establishment of preferential channels through the bed prevents a smooth desorption of the CO_2_. Besides this, the application of the sound also results in a faster heating rate, as clearly evidenced by the temperature profiles reported in Figure 10a, thanks to the larger heat transfer coefficients obtainable when the bed is fluidized, namely under sound-assisted conditions, with respect to those obtainable when the fluidization quality is poor, i.e., under ordinary conditions.

The beneficial effect derived by the application of the sound is also highlighted by evaluating some important desorption parameters, namely the recovery level, R, the desorption time at a fixed R, t_d_, and the average CO_2_ concentration C_m_. Clearly, as consequence of the peak-shaped desorption profile, whether the acoustic field is applied or not, higher t_d_ values result in higher regeneration efficiency, i.e., larger values of R. However, at the same time, larger values of t_d_ lead to lower values of C_m_, due to the increasing dilution effect caused by prolonged N_2_ purging. This is evidently shown in Figure 10b, where it is clear that, when t_d_ is increased, R monotonically increases and C_m_ monotonically decreases. It is also evident that, at each fixed value of t_d_, both R and C_m_ are always larger in the sound-assisted test than in the ordinary test.

**Figure 11:**
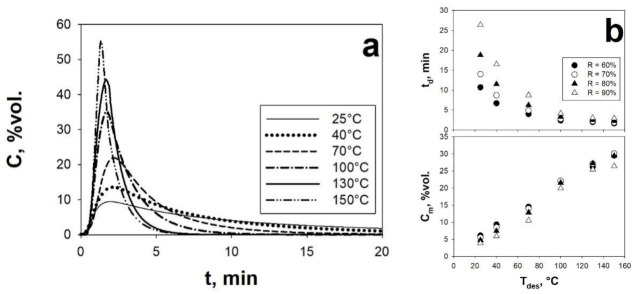
(**a**) Desorption peaks of AC Norit obtained, using the isothermal regeneration mode, under sound-assisted conditions at different T_des_ at a fixed Q_p_ (67.8 NL h^−1^). (**b**) t_d_ and C_m_ as functions of the T_des_ at fixed Q_p_ (67.8 NL h^−1^) and different values of R. Adsorption step: SPL = 140 dB; f = 80 Hz; Q_IN_ = 67.8 NL h^−1^; C_0_ = 10 vol.%. Adapted from [19] with permission.

**Figure 12:**
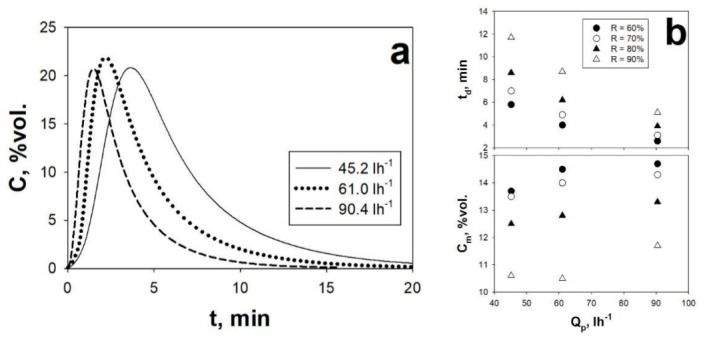
(**a**) Desorption peaks of AC Norit obtained, using the isothermal regeneration mode, under sound-assisted conditions at different Q_p_ at a fixed T_des_ (70 °C). (**b**) t_d_ and C_m_ as functions of Q_p_ at fixed T_des_ (70 °C) and different values of R. Adsorption step: SPL = 140 dB; f = 80 Hz; Q_IN_ = 67.8 NL h^−1^; C_0_ = 10 vol.%. Adapted from [19] with permission.

In analogy to the study performed on the CO_2_ adsorption step, the effect of some important operating variables on the CO_2_ desorption process, i.e., desorption temperature (T_des_) and N_2_ purge flowrate (Q_p_), has been also investigated in the SAFB, using the isothermal purging mode described in Section 2.3. The results obtained are shown in Figure 11 and Figure 12. First of all, it is important to highlight that, regardless of T_des_ and Q_p_, the sorbent can be fully regenerated (R = 100%), which agrees with the literature indicating the activated carbons as a physisorbent, i.e., the CO_2_ is adsorbed by the formation of weak physical bonds rather than strong chemical bonds on the sorbent surface [15,18].

With reference to the effect of T_des_, the analysis of Figure 11a highlights that the CO_2_ desorption process is fastened by increasing values of T_des_, as clearly denoted by the CO_2_ desorption peaks becoming higher and narrower as T_des_ is increased. This experimental evidence can be explained considering that at higher values of T_des_: (i) the thermodynamics equilibrium is shifted towards the desorption, namely the adsorption process is more and more penalized, meaning that the CO_2_ are more easily found in the desorbed rather than in the adsorbed state and (ii) the CO_2_ and N_2_ molecular diffusivities are increased. Then, based on the elaboration of the experimental desorption peaks, it has been highlighted in Figure 11b that: (i) the time required to achieve a certain value of R monotonically decreases with increasing T_des_ and (ii) at a fixed value of R, the concentration of the recovered CO_2_ stream can be remarkably improved increasing T_des_. Moreover, it is important to underline that, regardless of the N_2_ purge flow rates, values of T_des_ larger than 70 °C are enough to concentrate the captured CO_2_; indeed, as clearly shown in Figure 11b, C_m_ is larger than C_0_ (10 vol.%).

With reference to the effect of Q_p_, the analysis of Figure 12a evidences that the CO_2_ desorption process is fastened by increasing values of Q_p_, as evidently shown by the CO_2_ desorption peaks becoming narrower as Q_p_ is increased. However, differently from what observed with increasing T_des_, Q_p_ has no effect on the maximum of the desorption peak. As observed for T_des_, increasing values of Q_p_ also positively affect the desorption rate. Then, the analysis of Figure 11b shows that: (i) the time required to achieve a certain value of R monotonically decreases with increasing Q_p_ and (ii) the concentration of the recovered CO_2_ stream achievable at a fixed R is only slightly affected by Q_p_.

### 3.3. Calcium Looping (CaL)

**Figure 13:**
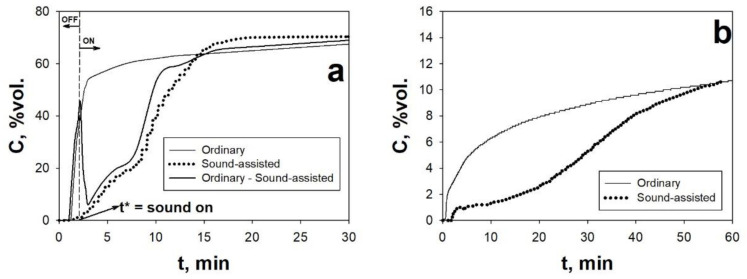
(**a**) Outlet CO_2_ concentration measured during carbonation in an ordinary fluidization test, a sound-assisted fluidization test and during a test in which the sound is switched on at t = t*. Tests performed in the SAFB for thermochemical energy storage (TCES) applications. Q_IN_ = 115 NL h^−1^; T = 850 °C; C_0_ = 70 vol.% (**b**) Outlet CO_2_ concentration measured during carbonation under ordinary and sound-assisted fluidization conditions. Tests performed in the Seville experimental apparatus for Carbon Capture and Storage (CCS) applications [37]. Q_IN_: 120 NL h^−1^; Carbonation: T = 650 °C; C_0_ = 15 vol.% Adapted from [5,37] with permission.

Since fine/ultrafine powders have been indicated as viable solution to overcome the limitation of the CaL, in the framework of both CCS and TCES application, the SAFB has been proposed in a CaL configuration (CaL-SAFB) as possible technology for the handling and processing of these materials. In particular, Figure 13a,b reports the results, in terms of time evolution of the outlet CO_2_ concentration, obtained in the SAFB, for TCES applications, and in the Seville experimental apparatus, for CCS applications, respectively.

It is clear that when the acoustic field is applied the carbonation performances are remarkably enhanced, regardless of the specific operating conditions of CCS and TCES. Indeed, the reaction degree, namely the quantity of CO_2_ and CaO reacted, is proportional to the area between the curve and the horizontal line corresponding to the inlet CO_2_ concentration. Clearly, under sound-assisted fluidization conditions, this area is bigger than under ordinary conditions, meaning that more CO_2_/CaO has reacted. More specifically, whether the acoustic perturbation is applied or not, the variation of the slope of the CO_2_ concentration profile clearly evidences the existence of the two stages of the carbonation reaction, namely a fast stage under kinetic control followed by a slow diffusion controlled one. However, the time at which the transition between these two stages occurs is strongly dependent on whether the sound is applied or not. In particular, under sound-assisted fluidization conditions the transition occurs much later than under ordinary conditions. Indeed, under ordinary conditions, the CaO is carbonated mainly in the slow stage under diffusion control, due to the relatively fast switch from the previous fast stage of the reaction. Under sound-assisted conditions, on the contrary, the contribution of the fast kinetically controlled stage to the global carbonation conversion is remarkably enhanced, thus resulting in a general speed-up of the process.

**Figure 14:**
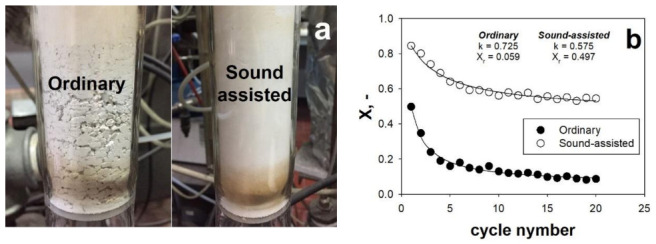
(**a**) Pictures of the fluidized bed taken during ordinary and sound-assisted fluidization tests in the SAFB; (**b**) evolution of the carbonation conversion with the cycle number and deactivation fit curve obtained under ordinary and sound assisted-fluidization conditions. T = 850 °C. Q_IN_: 115 NL h^−1^; C_0_ = 70 vol.%. Calcination: T = 750 °C; C_0_ = 0 vol.%. (i.e., pure N_2_). Adapted from [5] with permission.

These experimental evidences are consistent with the observed enhancement of the fluidization quality under sound-assisted fluidization conditions (Figure 3). Indeed, as also clearly shown in Figure 14a, the fluidization quality is particularly poor under ordinary conditions, i.e., characterized by severe channeling. As a consequence, when no sound perturbation is applied, the gas–solid contact efficiency is dramatically low: a large portion of both the gaseous, i.e., CO_2_, and solid, i.e., CaO, hardly takes part in the carbonation reaction due to gas by-passing, though channels, and solid agglomeration, which reduces the availability of free CaO surface. It is also evident from Figure 13 that the effect of the acoustic perturbation is stronger in the initial fast stage of the reaction, when the reaction is mainly due to the reaction of CO_2_ with the CaO surface and the sound can improve the gas–solid contact efficiency. On the contrary, the effect of the sound becomes less evident in the slow diffusion controlled stage, when the reaction is no longer controlled by the availability of free CaO surface and starts to be controlled by the diffusion of the CO_2_ molecules through the carbonate layer. Indeed, in this stage, the improved fluidization quality and gas–solid contact efficiency yielded by the acoustic perturbation have a negligible effect on the reaction.

It is also evident, from the results reported in Figure 14b, that the application of the acoustic field makes it possible to increase the residual carbonation conversion and reduce the rate of the natural deactivation of CaO due to sintering phenomena.

According to the literature [7,83,84], the progressive decrease of surface due to sintering leads to a dramatic loss of CaO multicyclic during cyclic operations. In particular, when performed under sound-assisted conditions, the fine CaO sorbent shows a residual conversion as high as X20 = 0.55 (X at the 20th cycle), which is remarkably higher than the value obtained with coarser limestone particles (X20 = 0.41 [7,47]) and also with CaO stabilized with sintering inhibitors (X20 = 0.46 [7,8]) at TCES-CSP operating conditions. This experimental evidence confirms that the employment of fine CaO particles can remarkably increase the carbonation performances, due to the hindrance of both sintering and pore-plugging phenomena. Besides this, it is also noteworthy that the results obtained using fine CaO particles in the SAFB are remarkably better than those reported for fine natural limestone tested in thermobalances [7,47]. This further confirms the capability of the SAFB configuration of getting the best performances out of fine CaO particles.

In regards to the calcination step, few calcination tests have been performed in the Seville experimental apparatus [37]. It was found that, even though the calcination of CaCO_3_ takes place at an already quick rate at temperatures as high as 900 °C under ordinary fluidization conditions, the application of sound makes it possible to accelerate it further.

## 4. Conclusions

The present manuscript presents the sound-assisted fluidized bed reactor (SAFB), designed and set-up in Naples. In particular, the main results obtained using the SAFB are reviewed, highlighting how the acoustic affects the fluid dynamics of the system and the performances/outcomes of the specific chemical processes performed therein.

The fluidization behavior of different nano-sized and micro-sized powders has been investigated. The application of an acoustic field, characterized by proper f and SPL, makes it possible to either achieve an appropriate fluidization regime or enhance the fluidization quality of the powders, regardless of the specific particle size of the tested materials. In particular, while under ordinary conditions the fluidization quality is particularly poor and heterogeneous, the bed is completely fluidized, with ideal-like fluidization curves and higher values of expansion ratio, when the acoustic perturbation is applied. The effect of the acoustic parameters, i.e., SPL and f, and of the temperature has been highlighted:

- Higher SPLs lead to a general enhancement of the fluidization quality (i.e., lower values of u_mf_ and d_agg_), whereas, an optimum range of frequency (providing minimum values of u_mf_ and d_agg_) can be found in the proximity of to the natural frequency of the powder.
- Increasing temperatures negatively affect the fluidization quality due to the intensification of IPFs. Regardless of the powders being A or C type, the fluidization parameters, u_mf_ and d_agg_, are increased when the temperature is increased.

The SAFB has been used in a TSA configuration (TSA-SAFB), demonstrating that fine/ultrafine powdered sorbents, regardless of their specific chemical nature, can be directly used as free-flowing powders in the TSA-SAFB also with a remarkable improvement of their adsorption/desorption performances with respect to their use in other standard reactor configurations (e.g., ordinary fluidized beds and fixed beds). The use of a proper acoustic field remarkably enhances both CO_2_ adsorption performances, in terms of higher values of equilibrium adsorption capacity (q_e_), BT time (t_b_) and fraction of bed utilized at t_b_ (ψ), and the CO_2_ desorption performances, in terms of higher values of CO_2_ recovery (R) and CO_2_ purity (C_m_), and shorter desorption times (t_d_). The effect of some important operating variables on the adsorption/desorption performances has been pointed out:

- Increasing values of the adsorption temperature (T_ads_) negatively affects the CO_2_ adsorption thermodynamics, i.e., higher values of T_ads_ lead to lower q_e_. On the contrary, increasing values T_ads_ positively affect the CO_2_ adsorption kinetics, i.e., higher values of T_ads_ lead to lower values of Δt.
- Increasing values of the CO_2_ partial pressure (P_CO_2__) positively affects the CO_2_ adsorption thermodynamics, i.e., higher values of P_CO_2__ lead to higher q_e_. Likewise, increasing values P_CO_2__ positively affect the CO_2_ adsorption kinetics, i.e., higher values of P_CO_2__ lead to lower values of Δt.
- Increasing values of the desorption temperature (T_des_) positively affects the CO_2_ desorption kinetics, i.e., for a fixed R, higher values of T_des_ lead to shorter t_d_ and higher C_m_.
- Increasing values of Q_p_ also positively affects the desorption rate, i.e., for a fixed R, higher values of Q_p_ lead to shorter t_d_ and higher C_m_.

The SAFB has been proposed in a CaL configuration, CaL-SAFB, as a possible technology for the handling and processing of fine/ultrafine CaO/CaCO_3_ particles aiming at overcoming the shortcomings/limits of the standard CaL, in the framework of both CCS and TCES application. The results obtained shows that, when the acoustic field is applied, the carbonation performances are remarkably enhanced, regardless of the specific operating conditions of CCS and TCES. In particular:

- The beneficial effect of the acoustic perturbation is much stronger in the early fast stage of the carbonation, when the reaction is mainly governed by the reaction of CO_2_ with the CaO surface and the sound can improve the gas–solid contact efficiency.
- The acoustic perturbation also leads to the increase of the residual carbonation conversion and to the reduction of the rate of the natural deactivation of CaO due to sintering phenomena.
- When performed under sound-assisted conditions, the fine CaO particles can provide a residual conversion remarkably higher that the values reported for coarser limestone and even for CaO stabilized with sintering inhibitors.

## Figures and Tables

**Table 1:** Materials used in the SAFB.

Dimension	Name	Primary Particle Size	D_sauter_ (μm)	ρ_p_ (kg m^−3^)	BET SSA (m^2^ g^−1^)	V_p_ (cm^3^ g^−1^)	m (g)	Ref.
**Nano-sized**	Al_2_O_3_	<50 nm	2.02	4000	-	-	48	[55,66,67]
	Fe_2_O_3_	<50 nm	0.43	4500	-	-	33	[55,66,67]
	ZrO_2_	<50 nm		6000	-	-	35	[55]
	CuO	<50 nm		6300	-	-	75	[55]
**Micro-sized**	AC Norit	-	0.39	-	1060	1.34	100	[3,4,15,19,20,21,37,55]
	AC Sigma	-	15.40	-	1038	1.14	100	[3]
	HKUST-1	-	4.30	-	680	0.66	50	[3,68]
	Ashes	<60 μm	8.00	2000	-	-	-	[54]
	Silica sand	<150 μm	60.00	2600	-	-	-	[54]
	Natural CaCO_3_	-	4.00	-	1.60	-	100	[5,6]
	* Synthetic CaO	-	<100	-		-	100	[37]

ρ_p_ = particle density; BET SSA = BET specific surface area; V_p_ = pore volume; m = mass of powder loaded in the SAFB. * Tests performed in the Seville experimental apparatus.

**Table 2:** Results of the ordinary and sound-assisted CO_2_ adsorption tests.

Type of Test	^1^ AC Norit [4]			^2^ HKUST-1 [68]		
	q_e_ mmol g^−1^	t_b_ s	ψ -	q_e_ mmol g^−1^	t_b_ s	ψ -
Ordinary	0.31	12	2.7	0.78	26	8
Sound-assisted1	0.37	63	15	1.14	141	29

^1^ SPL= 140 dB; f = 80 Hz; P_CO_2__ = 0.10 atm; u = 1.5 cm/s; T_ads_ = ambient temperature. ^2^ SPL= 140 dB; f = 120 Hz; P_CO_2__ = 0.15 atm; u = 1.5 cm/s; T_ads_ = ambient temperature.
